# Connection between oral health and chronic diseases

**DOI:** 10.1002/mco2.70052

**Published:** 2025-01-14

**Authors:** Di Fu, Xingyue Shu, Ge Zhou, Mengzhen Ji, Ga Liao, Ling Zou

**Affiliations:** ^1^ State Key Laboratory of Oral Diseases, National Center for Stomatology, National Clinical Research Center for Oral Diseases Sichuan University Chengdu Sichuan China; ^2^ Department of Information Management, Department of Stomatology Informatics, West China Hospital of Stomatology Sichuan University Chengdu Sichuan China; ^3^ State Key Laboratory of Oral Diseases, National Center for Stomatology, National Clinical Research Center for Oral Diseases, Department of Conservative Dentistry and Endodontics, West China Hospital of Stomatology Sichuan University Chengdu Sichuan China

**Keywords:** cardiovascular diseases, chronic diseases, chronic respiratory diseases, diabetes, oral health, rheumatoid arthritis

## Abstract

Chronic diseases have emerged as a paramount global health burden, accounting for 74% of global mortality and causing substantial economic losses. The oral cavity serves as a critical indicator of overall health and is inextricably linked to chronic disorders. Neglecting oral health can exacerbate localized pathologies and accelerate the progression of chronic conditions, whereas effective management has the potential to reduce their incidence and mortality. Nevertheless, limited resources and lack of awareness often impede timely dental intervention, delaying optimal therapeutic measures. This review provides a comprehensive analysis of the impact of prevalent chronic diseases—such as diabetes mellitus, rheumatoid arthritis, cardiovascular disorders, and chronic respiratory diseases—on oral health, along with an exploration of how changes in oral health affect these chronic conditions through both deterioration and intervention mechanisms. Additionally, novel insights into the underlying pathophysiological mechanisms governing these relationships are presented. By synthesizing these advancements, this review aims to illuminate the complex interrelationship between oral health and chronic diseases while emphasizing the urgent need for greater collaboration between dental practitioners and general healthcare providers to improve overall health outcomes.

## Graphical abstract

Mechanisms linking oral health to chronic diseases. The figure highlights oral health connections to diabetes, cardiovascular diseases, rheumatoid arthritis, and respiratory diseases. Key mechanisms include inflammation, microbial dysbiosis, oxidative stress, and immune dysregulation, emphasizing the systemic impacts of oral diseases like periodontitis.

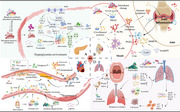

## 1 INTRODUCTION

Noncommunicable diseases (NCDs), including cardiovascular diseases (CVDs), diabetes mellitus (DM), chronic respiratory diseases, cancers, and obesity, are characterized by their prolonged nature and often incurability. According to the World Health Organization (WHO), NCDs account for 74% of global mortality, with approximately 17 million individuals succumbing before the age of 70 each year.^1^ These diseases impose a substantial economic burden, with healthcare expenditures surpassing 85% of total global healthcare costs.^2^ In the US healthcare system alone, over one trillion dollars are allocated annually to the management of chronic diseases.^3^ By 2030, the economic burden of major NCDs is projected to result in a loss of output amounting to $47 trillion, equivalent to 75% of the global gross domestic product (GDP) in 2010.^4^ Moreover, aging populations and declining birth rates are anticipated to further exacerbate this burden.

The oral cavity, situated at the intersection of medicine and dentistry, serves as a critical indicator of overall health, with its pathological conditions frequently exerting a more profound impact on systemic health than is commonly acknowledged by many healthcare providers.^5^ Ignoring oral health can not only exacerbate localized issues but also have significant repercussions on chronic diseases.^6^, ^7^, ^8^, ^9^ Proactively managing oral health through regular dental examinations and the maintenance of proper oral hygiene can significantly enhance quality of life while aiding in the prevention and management of chronic diseases, thereby reducing their incidence and mortality rates.^10^, ^11^, ^12^, ^13^ Regrettably, due to limited access to medical resources and dental care,^14^ many patients only recognize the close association between oral and systemic health after experiencing more severe health events, which subsequently delays optimal treatment and prevention. It is imperative to further explore this domain to enhance awareness of the critical role that oral health plays in the development of chronic diseases and to underscore its significance in the prevention and management of systemic conditions.

**FIGURE 1:**
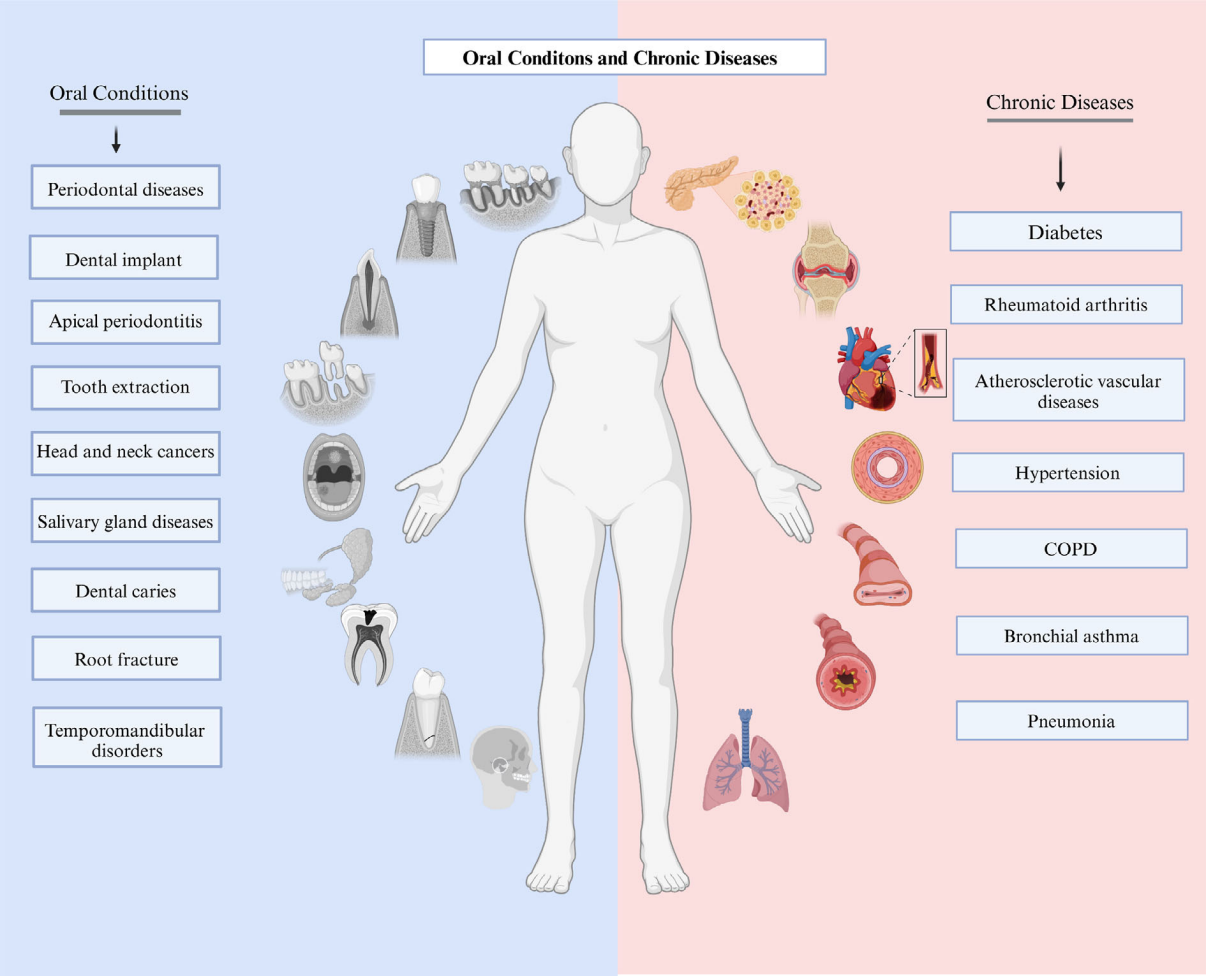
Oral conditions and chronic diseases. The left portion of the chart presents the common oral conditions referred to in this article, while the right portion exhibits the chronic diseases closely associated with oral conditions. Created with BioRender.com.

This review seeks to elucidate the relationships between oral health and prevalent chronic diseases (Figure 1), including DM, rheumatoid arthritis (RA), CVDs, and chronic respiratory diseases. It highlights recent advancements in understanding the mechanisms that connect oral health to these ailments and discusses strategies for enhancing oral care while managing chronic diseases.

## 2 DIABETES MELLITUS AND ORAL HEALTH

DM encompasses a spectrum of metabolic disorders characterized by insufficient insulin secretion, insulin resistance, or a combination of both, leading to impaired regulation of blood glucose levels. It exhibits a complex and interdependent relationship with oral health. Hyperglycemia increases the risk of oral diseases, including periodontal diseases, apical periodontitis (AP), head and neck cancer (HNC), salivary gland diseases, dental caries, and root fracture, while also impeding the healing process of extraction sites. Glycemic control is intricately associated with the success rates of dental implants and root canal treatment (RCT) in patients with diabetes. Moreover, declining oral health conditions, such as periodontal disease, can increase the prevalence of diabetes and negatively impact glycemic control. Furthermore, diabetic patients frequently experience salivary gland dysfunction and chronic inflammation, resulting in complications such as xerostomia, infections, and oral mucosal lesions.

Consequently, the maintenance of oral health should be considered a vital component of comprehensive diabetes management to mitigate the incidence of related complications and enhance patients' quality of life. To facilitate readers' access to pertinent literature, we have summarized recent reviews, systematic evaluations, and meta‐analyses regarding the relationship between diabetes and various oral diseases in the table (Table 1).

**TABLE 1:** Recent reviews/systematic reviews/meta‐analyses on diabetes and oral diseases.

Topic	Author, year	Study type	Main contents
Overall oral health	Alqadi, 2024^15^	R	The oral manifestations of DM are delineated, along with essential dental considerations for the management of diabetic patients during treatment.
	Grisi, 2022^16^	R	The authors examine prevalent oral signs and symptoms of diabetes, highlighting the mechanisms that connect DM to oral diseases and the associated risks of inadequate oral health in patients with DM.
	Ahmad, 2021^17^	R	The relationship between DM and oral complications is reviewed.
	Borgnakke, 2021^18^	R	The relationship between oral diseases and DM, including the underlying mechanisms, is summarized. Additionally, prevalent risk factors are delineated.
	Negrini, 2021^19^	R	The principal oral manifestations of DM are summarized. The interactions among diabetes, its risk factors, the oral microbiome, and oral diseases are analyzed.
Periodontitis	Enteghad, 2024^20^	R	Epidemiological and clinical evidence regarding the association between DM and periodontitis is summarized. The predictive value of periodontal parameters for identifying diabetes is emphasized, and the biological mechanisms through which diabetes induces periodontal disease are reviewed.
	Mirnic, 2024^21^	R	The clinical and epidemiological studies examining the bidirectional relationship between DM and periodontal disease, along with the pathogenesis and the role of oxidative stress, have been succinctly summarized.
	Vlachou, 2024^22^	R	The bidirectional relationship between DM and periodontitis is investigated, with a focus on the cellular and molecular mechanisms underlying the interaction between the oral microbiome and the host immune response.
	Păunică, 2023^23^	R	The most recent findings regarding the etiological factors, treatment, and prevention of both diseases are presented, with an emphasis on the role of diabetic microvascular complications, the oral microbiome, and inflammatory mediators.
	Zhao, 2023^24^	R	The potential mechanisms underlying the interaction between periodontitis and diabetes are summarized into six points: (1) microbiome factors; (2) enhanced inflammatory responses through cytokines, adipokines, AGE/RAGE, and miRNAs; (3) host immune factors; (4) oxidative stress; (5) alveolar bone resorption damage; and (6) epigenetic changes.
	Nibali, 2022^25^	R	The comorbidities associated with DM and periodontitis are summarized, and the underlying mechanisms are examined.
	Salhi, 2022^26^	R	The data concerning the impact of periodontitis on diabetes and vice versa are summarized in a descriptive table.
	Genco, 2020^27^	R	The role of diabetes as a risk factor for periodontal and other oral diseases is reviewed.
	Genco, 2020^28^	R	Studies elucidating the adverse effects of periodontal disease and diabetes are discussed, with a summary of their implications for clinical practice and public health.
	Graves, 2020^29^	R	The impact of DM on periodontal tissues, including periodontal ligament cells, osteoblasts, and osteocytes, is summarized. Furthermore, the effects of diabetes on the oral microbiome and its role in exacerbating periodontal bone loss are discussed.
	Polak, 2020^30^	R	The progression from DM to periodontitis is delineated, with a focus on microbiome alterations and immune changes in poorly controlled diabetes. Both microbiological and immunological shifts contributing to this condition are summarized.
	Costa, 2023^31^	S	The prevalence and severity of periodontal disease are higher in individuals with T1DM compared to the healthy population.
	Maia, 2023^32^	S	Fewer than half of diabetic patients are aware of their elevated risk for periodontal disease, and dentists frequently do not serve as the primary source encouraging them to seek this information.
	Nguyen, 2020^33^	S	Patients with periodontal disease are at an increased risk of developing diabetes‐related complications.
	Stöhr, 2021^34^	SM	There exists a positive bidirectional association between periodontitis and DM, underscoring the necessity of screening diabetic patients for periodontitis and vice versa.
	Baeza, 2019^35^	SM	SRP significantly affects metabolic control and reduction of systemic inflammation in patients with T2DM.
Dental implants	Zhang, 2023^36^	R	The structure of peri‐implant soft tissue sealing, associated diseases and their treatments, as well as the regulatory mechanisms of T2DM in relation to soft tissue sealing damage, are comprehensively reviewed.
	Vijay, 2021^37^	R	Recent advancements in the application of carbon‐based nanomaterials for enhancing dental implants in diabetic patients are summarized.
	Bencze, 2024^38^	SM	Within the constraints of this study, patients with poorly controlled T2DM or prediabetes may exhibit worse peri‐implant conditions compared to individuals without diabetes and those with well‐controlled T2DM. Furthermore, well‐controlled T2DM does not serve as a risk indicator for peri‐implant diseases.
	Al Ansari, 2022^39^	SM	Implants in diabetic patients exhibit a higher risk of failure and increased marginal bone resorption compared to nondiabetic individuals, with T1DM demonstrating a greater failure rate than T2DM.
	Andrade, 2022^40^	SM	With adequate blood glucose control, optimal oral hygiene, and strict adherence to procedural protocols, T2DM does not present a risk for immediate loading of implants.
	Lv, 2022^41^	SM	Diabetes or hyperglycemia upregulates negative regulators of bone metabolism, harming peri‐implant health. HbA1c levels are dose related to advanced glycation end products, periodontal disease, and marginal bone loss.
	Shang, 2021^42^	SM	BOP and PIBL were significantly higher in T2DM patients. However, strict oral hygiene reduced the impact of blood sugar levels on these parameters.
AP	Tibúrcio‐Machado, 2017^43^	R	Current literature suggests a positive correlation between diabetes and an increased prevalence of periapical lesions.
	Pérez‐Losada, 2020^44^	SM	There may be common pathophysiological factors underlying both AP and DM; however, the relationship between these two conditions necessitates further prospective studies.
	Liu, 2023^45^	SM	DM may elevate the risk of AP in teeth that have undergone endodontic treatment. Furthermore, in cases where apical periodontitis is already established, DM may exacerbate the progression of this condition.
Head and neck cancer	Wang, 2020^46^	R	Recent advancements in the research concerning the relationship between DM and the mechanisms underlying head and neck cancer development have been summarized.
	Ramos‐Garcia, 2021^47^	SM	The prevalence and risk of oral cancer, as well as potentially malignant oral diseases, are elevated in individuals with DM, and their associated mortality rate from oral cancer is significantly higher than that of nondiabetic individuals.
	Yan, 2021^48^	SM	T2DM is associated with an increased risk of head and neck cancers in East Asia.
Salivary	Pérez‐Ros, 2021^49^	R	Salivary amylase and glucose concentrations represent potential noninvasive biomarkers for evaluating blood glucose control and clinical management in patients with DM.
Dental caries	Zhou, 2024^50^	SM	T2DM may lead to an increased dental caries index in adults, along with reduced salivary flow rate, pH, and buffering capacity.
	Weijdijk, 2023^51^	SM	Compared to nondiabetic patients, diabetic patients have a higher DMF index score with moderate certainty.
	Coelho, 2020^52^	SM	T1DM patients have a higher risk of dental caries.
	de Lima, 2020^53^	SM	DM may increase the incidence of coronal and root caries in adults. Poor glycemic control makes diabetic patients more susceptible to dental caries.

Abbreviations: AGE, advanced glycation end product; AP, apical periodontitis; BOP, bleeding on probing; DM, diabetes mellitus; PIBL, peri‐implant bone loss; R, review; RAGE, receptor for advanced glycation end product; S, systematic review; SM, systematic and meta‐analysis; SRP, scaling and root planning; T1DM, type 1 diabetes mellitus; T2DM, type 2 diabetes mellitus.

### 2.1 Diabetes and periodontal diseases

Robust epidemiological and clinical evidence substantiates the association between the two.^20^, ^21^, ^22^ Diabetic patients exhibit a significantly higher prevalence of periodontitis and show worse periodontal parameters, including clinical attachment loss, periodontitis surface area, and the number of teeth lost.^20^, ^54^, ^55^, ^56^, ^57^, ^58^ Conversely, individuals with periodontitis are at an increased risk of developing diabetes.^6^

**FIGURE 2:**
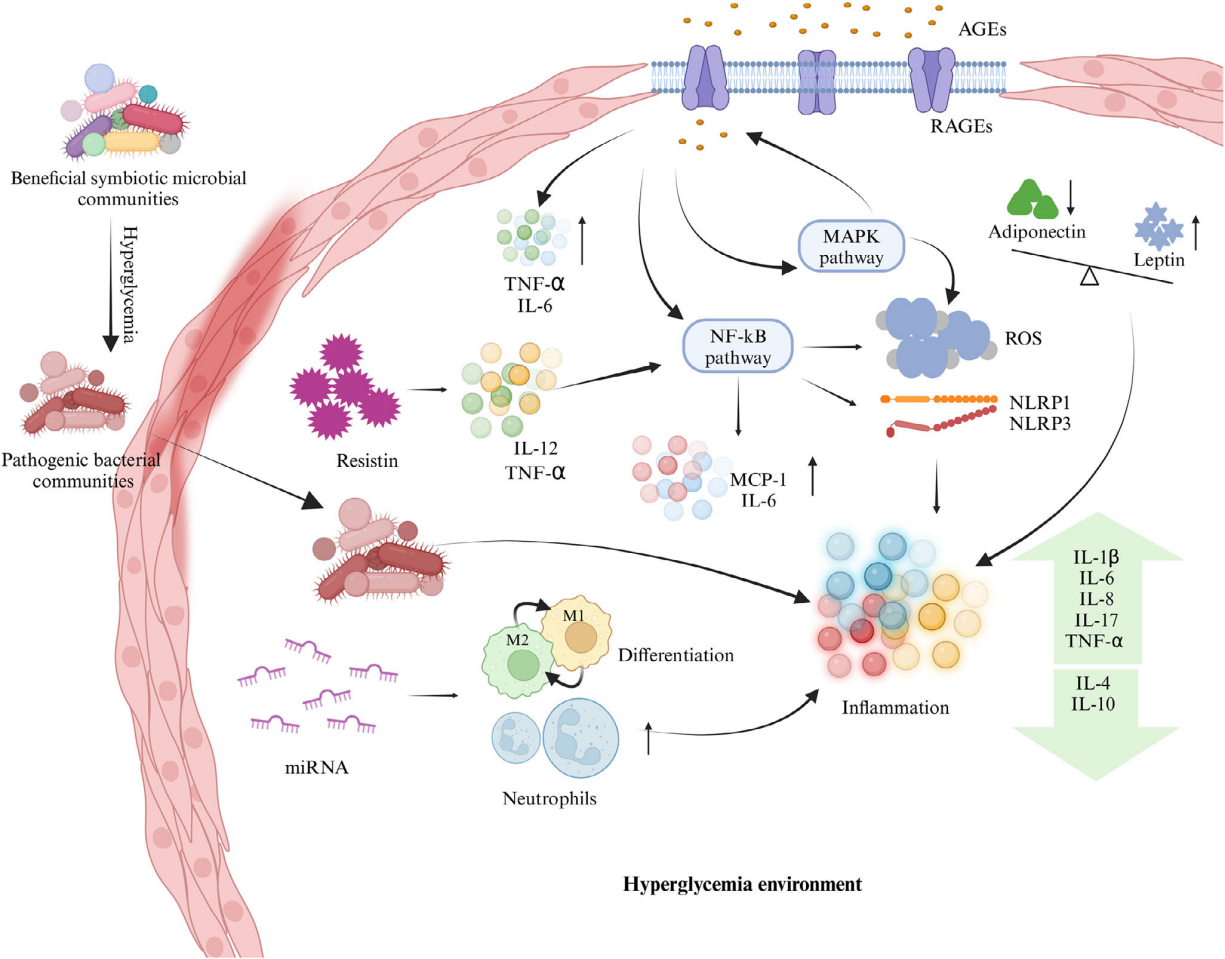
Mechanisms linking oral health and diabetes. Diabetes induces oral inflammation through multiple pathways, transforming beneficial microbial communities into pathogenic ones. Additionally, AGEs and resistin elevate inflammatory cytokines, thereby activating classical inflammatory pathways and generating ROS and inflammasomes. Dysregulation of adiponectin, leptin, and miRNA expression further drives immune cell differentiation and recruitment, exacerbating inflammation. AGEs, advanced glycation end products; IL‐4, interleukin‐4; IL‐6, interleukin‐6; IL‐8, interleukin‐8; IL‐10, interleukin‐10; IL‐17, interleukin‐17; MCP‐1, monocyte chemoattractant protein‐1; MMP, matrix metalloproteinase; M1, M1 macrophage; M2, M2 macrophage; NLRP1, NLR family pyrin domain containing 1; NLRP3, NLR family pyrin domain containing 3; RAGEs, receptor for advanced glycation end products; ROS, reactive oxygen species; TNF‐α, tumor necrosis factor‐alpha. Created with BioRender.com.

A recent study delineated the pathophysiological mechanisms through which diabetes impacts periodontal disease, encompassing vascular alterations in the gingiva, immune responses, collagen metabolism disorders, and specific microbiological profiles within periodontal pockets. Similarly, the mechanisms by which periodontal disease may affect diabetes include increased serum oxidative stress markers, elevated inflammatory markers, and enhanced insulin resistance.^21^ In 2023, Zhao et al. identified six potential mechanisms underlying the interaction between periodontitis and diabetes (Figure 2): (1) microbial factors; (2) enhanced inflammatory response through cytokines, adipokines, advanced glycation end product/receptor for advanced glycation end product (AGE/RAGE) pathways, and miRNAs; (3) host immune factors; (4) oxidative stress; (5) alveolar bone resorption damage; and (6) epigenetic changes.^24^ Later, Enteghad et al. added that the uncoupling of resorptive and formative responses in connective tissue.^20^ To provide a novel perspective on the interaction between diabetes and periodontitis, we reviewed the literature pertaining to the underlying mechanisms (Table 2) utilizing key molecules that may play significant roles in both diseases as indicators.

**TABLE 2:** Key molecules in the interaction between diabetes and periodontitis.

Core factors	Main findings	Clinical study	In vivo	In vitro
IL‐1 beta	Inflammatory cytokines play a role in the interplay between diabetes and periodontitis			^62^
IL‐6		^63^	^64^	
IL‐10		^65^		
IL‐12			^66^	
IL‐17			^67^, ^68^	
IL‐21		^69^		
TNF‐E		^65^, ^70^, ^71^	^72^, ^73^, ^74^, ^75^	^76^
Adiponectin	Adiponectin has anti‐inflammatory effects and is linked to diabetes and glycemic control. After periodontal therapy, increased adiponectin can improve insulin resistance and lower HbA1c. The adiponectin receptor agonist, AdipoAI, may reduce tissue inflammation and damage, suggesting potential for treating diabetes‐related periodontitis.	^77^	^78^	
Visfatin	Periodontal inflammation can upregulate proinflammatory cytokines such as IL‐6 and IL‐1b, leading to elevated visfatin expression in periodontal tissues. Visfatin may play a role in the pathogenesis of periodontitis by promoting the upregulation of matrix metalloproteinase‐1 and chemokine CC‐motif ligand 2 in periodontal ligament cells.	^79^		
ROS	ROS production induces MAPK and NLRP3‐related protein expression in high glucose conditions. ROS act as upstream signals for MAPKs, NF‐kappa B, and the NLRP3 inflammasome, leading to their activation. This process, along with increased oxidative damage and downregulation of Nrf2, contributes to the onset and progression of diabetic periodontitis.	^58^		^62^, ^76^, ^80^
MAPK				^80^
NLRP3				^80^
NF‐κB			^81^	^80^, ^81^
Nrf2			^82^	
MMP	Elevated levels of MMP‐8 and MMP‐9 are associated with more severe periodontitis and an increased risk of diabetes mellitus.	^56^, ^83^		
CRP	CRP affects periodontitis and diabetes by inhibiting osteoblast formation and promoting osteoclast formation through the PI3K/AKT signaling pathway, disrupting alveolar bone homeostasis.	^84^	^85^	
hBDs	The concentrations of hBD‐2 and hBD‐3 are closely related to periodontal health and diabetic status.	^86^, ^87^		
GLUT1	GLUT1, a key glucose transporter in macrophages, is upregulated in the gingiva of diabetic mice. It contributes to high glucose‐induced macrophage senescence and the SASP response, potentially worsening diabetic periodontitis.		^88^	
AGEs	In chronic hyperglycemic conditions, AGEs significantly increase and affect periodontal tissue cell function by binding to RAGE, correlating with the severity of periodontitis. Additionally, AGEs negatively impact the skeletal system by inhibiting the expression of osteoblast‐related molecules, exacerbating periodontal disease in diabetic patients.	^70^, ^89^	^73^	^76^, ^90^, ^91^, ^92^, ^93^
WBC	WBCs worsen the development and progression of periodontitis in diabetic patients by amplifying inflammatory responses and impairing immune function.	^84^, ^94^		
PMNs	The systemic effects of periodontal tissue inflammation may intensify the innate immune response of neutrophils, promoting the interaction between diabetes and periodontitis.		^95^	
RANKL	Oral infections and diabetes can upregulate osteoclast RANKL expression, increasing the number and activity of osteoclasts, which leads to bone loss. In diabetic patients, RANK and RANKL expression are elevated, while OPG expression is reduced.		^96^	
Microbial change	Diabetes can alter the diversity and composition of the oral microbiome, particularly the subgingival microbiota.	^97^, ^98^, ^99^		
Gut microorganism	In PD patients, alterations in gut microbiota composition, particularly the reduction of butyrate‐producing bacteria, are associated with elevated blood glucose levels and impaired glucose tolerance. Using antibiotics or fecal microbiota transplantation to eliminate gut bacteria can improve blood glucose levels and inflammation markers.		^100^	
miRNA	miR‐146a and miR‐155 may be novel biomarkers for periodontitis in both diabetic and nondiabetic patients. Additionally, miR‐126, by targeting TRAF6 and reducing inflammation in gingival fibroblasts under high glucose conditions, could serve as a potential therapeutic target for treating periodontitis in diabetic patients.	^101^	^102^, ^103^	^103^, ^104^, ^105^, ^106^
SNPs	SNPs influence disease susceptibility and severity. SNPs in the IL‐1, IL‐4, and IL‐6 genes are significantly associated with the risk of diabetes and periodontitis.	^107^		
25VD_3_	Patients with diabetes and periodontitis have lower levels of vitamin D and calcium, which are negatively correlated with RBS and HbA1c levels.		^108^	

Abbreviations: AGEs, advanced glycation end products; CRP, C‐reactive protein; GLUT1, the glucose transporter 1; hBDs, human beta‐defensins; IL, interleukin; MAPK, mitogen‐activated protein kinase; MMP, metalloproteinase; NF‐κB, nuclear factor kappa‐B; NLRP3, NOD‐like receptor pyrin domain containing three; Nrf2, nuclear factor erythroid 2‐related factor 2; OPG, osteoprotegerin; PD, periodontal diseases; PMNs, polymorphonuclear neutrophils; RAGEs, receptor for advanced glycation end products; RANK, receptor activator of nuclear factor‐κ B; RANKL, receptor activator of nuclear factor‐κ B ligand; RBS, random blood sugar; ROS, reactive oxygen species; SASP, senescence‐associated secretory phenotype; SNP, single nucleotide polymorphisms; TNF‐α, tumor necrosis factor‐alpha; VD, vitamin D; WBC, white blood cell.

With the advancement of various saliva‐based methodologies, markers, and models for the clinical screening of diabetes and prediabetes, saliva has emerged as a promising medium for this purpose. Dentists may leverage noninvasive chairside techniques to screen or diagnose diabetes and collaborate effectively with internists to manage the oral health of diabetic patients. Studies have demonstrated that placental growth factor (PlGF)^59^ in gingival crevicular fluid (GCF) and microbial components^60^ in saliva are correlated with blood glucose levels. These findings offer a promising avenue for the noninvasive auxiliary diagnosis of DM. In addition, researchers have developed diagnostic models capable of screening for prediabetes and diabetes in patients with periodontitis,^61^ demonstrating moderate to good discriminative ability. In the future, dental clinics may assume an increasingly vital role in diabetes screening.

### 2.2 Diabetes and dental implants

Dental implants represent an effective solution for the replacement of missing teeth; however, their success is contingent upon both the local periodontal conditions and the overall health of patients, particularly with respect to a history of diabetes. Ongoing discussions examine how diabetes influences dental implant outcomes, with some researchers suggesting that diabetes may lead to complications surrounding implants and impede bone healing, ultimately resulting in a lower surgical success rate.^109^, ^110^, ^111^ Even if the initial success rates for implants in diabetic individuals can be quite high, the long‐term results often seem less promising.^112^ Conversely, other studies report differing results, indicating that the implant success rate remains within an acceptable range.^113^, ^114^, ^115^, ^116^, ^117^, ^118^ This inconsistency may stem from variations in diagnostic criteria and other influencing factors, particularly differences in the glycemic control of the studied patients.^38^, ^39^, ^40^, ^42^, ^119^ The evidence derived from two recently published meta‐analyses indicates that the management of blood glucose levels is a critical factor influencing the success of implants in diabetic patients.^41^, ^120^

The mechanisms through which diabetes influences dental implants are analogous to its effects on osteogenesis and osteoclastogenesis.^121^ A review conducted by de Oliveira et al. delineated these mechanisms as follows: (1) disrupting the balance between bone resorption and synthesis; (2) inducing microvascular disease; and (3) diminishing markers of bone formation while affecting collagen structure.^122^ Furthermore, AGEs‐related NOX (NADPH oxidases)‐mediated oxidative stress significantly impairs bone healing in diabetic patients.^123^

Future improvements in the success rates of dental implants for diabetic patients are expected to focus on enhancing implant material properties,^37^ refining implant coatings,^124^ and advancing surgical techniques.^125^

### 2.3 Diabetes and apical periodontitis

AP manifests at the apex of the tooth root, leading to inflammation in both dental and periodontal tissues. Root canal therapy (RCT) serves as the primary treatment modality. Although evidence remains limited, existing studies indicate a positive correlation between diabetes and the prevalence of AP lesions.^43^ Poor blood glucose control can lead to higher rates of AP and increased failure rates of RCT.^126^, ^127^, ^128^, ^129^ The impact of RCT on blood glucose management in diabetic patients is still under debate.^130^, ^131^, ^132^ The discrepancies in findings may be attributed to differences in criteria, limited sample sizes, or varying follow‐up periods. Further well‐structured, large‐scale clinical trials are needed.

Existing evidence suggests that diabetes may exacerbate both the incidence and progression of AP by modulating levels of inflammatory factors, oxidative stress, and the prevalence of *Candida albicans* (*C. albicans*) within periodontal tissues. Several animal studies have underscored the significant roles of interleukin‐6 (IL‐6), tumor necrosis factor‐alpha (TNF‐α), and interleukin‐17 (IL‐17) in the pathogenesis and progression of both diabetes and AP.^68^, ^133^ Furthermore, diabetes is known to disrupt the antioxidant balance by elevating malondialdehyde (MDA) and uric acid levels while concurrently decreasing albumin concentrations. The presence of AP exacerbates these diabetic effects, leading to further reductions in albumin and increases in uric acid levels.^134^ Additionally, another investigation demonstrated that the presence of *C. albicans* in type 2 diabetes mellitus (T2DM) individuals correlates with a higher incidence of AP.^135^

### 2.4 Diabetes and tooth extraction

Persistent hyperglycemia and chronic inflammation associated with DM can adversely affect the healing process of extraction sockets.^136^, ^137^, ^138^ This underscores the importance of blood glucose control for diabetic patients undergoing tooth extraction. However, many oral surgeons remain uncertain about the critical thresholds for blood glucose and HbA1c levels, as well as the point at which the risk of adverse complications significantly escalates.^139^ A systematic review has provided new insights, indicating that a fasting blood glucose level of 240 mg/dL serves as the critical threshold for any dental treatment, as warning signs of diabetes begin to manifest at this level. For diabetic patients, the maximum acceptable blood glucose levels prior to tooth extraction are 180 mg/dL before meals and 234 mg/dL 2 h postprandially.^140^ Moreover, the use of prophylactic antibiotics is not sufficiently supported by evidence. T2DM does not necessitate prophylactic antibiotics prior to tooth extraction in the absence of acute odontogenic infections.^138^ A systematic review indicates that there is currently no scientific evidence to substantiate the efficacy of prophylactic antibiotic use prior to dental surgery in patients with diabetes.^141^

The characteristics of diabetic wounds encompass elevated levels of ROS, impaired M1/M2 macrophage polarization, and chronic inflammation driven by proinflammatory chemokines.^139^ The challenges associated with healing postextraction wounds are linked to diminished osteogenic differentiation of mesenchymal stem cells (MSC),^142^ activation of matrix metalloproteinase‐9 (MMP‐9), persistent imbalance in the receptor activator of nuclear factor‐κ B ligand (RANKL)/osteoprotegerin (OPG) ratio.^143^ Furthermore, the hyperglycemic environment disrupts the secretion of cytokines such as TNF‐α, IL‐6, and IL‐1β from macrophages,^144^ resulting in functional impairments in neutrophils during the inflammatory response stages of wound healing, including migration, chemotaxis, and adhesion.^138^

### 2.5 Diabetes and head and neck cancer

HNCs encompass a range of malignancies originating in regions such as the oral cavity, pharynx, larynx, paranasal sinuses, nasal cavity, salivary glands, and lymph nodes within the head and neck area.^145^ Among these, cancers directly impacting the oral cavity and adjacent areas, such as oral squamous cell carcinoma (OSCC) and oropharyngeal carcinoma, are of particular concern due to their profound implications for oral health and systemic well‐being. Besides, for advanced‐stage cancers, the application of high‐energy radiation can lead to various potentially severe oral complications, including reduced salivary secretion causing xerostomia, oral pain, bacterial and fungal infections, taste disturbances, swallowing difficulties, discomfort, bleeding of periodontal tissues, and speech impairments.^146^, ^147^ This section focuses on the relationship between the aforementioned cancers that directly affect oral health and chronic diseases, as well as the impact of cancer treatments and medications on chronic conditions.

DM has been associated with an increased risk of developing various cancers, including HNC.^46^, ^47^, ^148^ T2DM promotes proliferation, metastasis, and inhibits apoptosis in OSCC.^149^ Patients with oral and oropharyngeal cancers who have a history of diabetes may have lower survival rates.^150^

Researches indicate that antidiabetic medications such as metformin,^46^ pioglitazone,^151^ and SGLT‐2 inhibitors^152^, ^153^ exhibit antitumor effects. A review has elucidated the mechanisms of action of SGLT‐2 inhibitors and their applications in the management of type 2 diabetes and its associated complications. Both SGLT1 and SGLT2 are expressed in various tumors, where they facilitate euglycemic glycolysis by supplying tumor cells with glucose. Furthermore, the proliferation of carcinomas expressing SGLT2 can be effectively inhibited through the administration of an SGLT2 inhibitor.^154^ Besides, the effects ofGLP‐1 receptor agonists (GLP‐1RAs) on tumors remain ambiguous, with some studies suggesting a potential increase^155^, ^156^ in cancer risk and others indicating otherwise.^157^, ^158^ Additional experiments are warranted to investigate.

The association between diabetes and HNC may be attributed to shared risk factors inherent to both conditions.^159^ A review published in 2020 delineated the potential mechanisms by which diabetes may influence the occurrence, metastasis, and prognosis occ. These mechanisms include hyperglycemia, hyperinsulinemia, insulin resistance, chronic inflammation, and immune dysfunction.^46^

### 2.6 Diabetes and salivary gland diseases

In recent years, accumulating evidence has established a robust association between salivary gland diseases and diabetes.^160^ Salivary gland dysfunction in diabetic patients is predominantly characterized by impaired salivary secretion, resulting in diminished saliva flow, a sensation of dry mouth, and an elevated risk of dental caries.^161^, ^162^ Considering that the management of xerostomia should prioritize addressing its underlying causes,^163^ effective blood glucose control is essential for diabetic patients experiencing this condition. When indicated, local saliva substitutes and/or stimulants may be utilized as adjunctive therapies.

Nitric oxide synthase (NOS) and its cofactor tetrahydrobiopterin (BH4) have been identified as promising targets for mitigating the reduced salivary secretion associated with diabetes‐induced xerostomia.^164^ Immunohistochemical analyses of diabetic rats further elucidate that inflammation and oxidative stress are critical contributors to the progression of this condition.^165^ Additionally, acinar cell vacuolation,^166^ intracellular structural damage,^167^ and dysfunction of the cholinergic vasodilation pathway^168^ have been implicated as potential mechanism.

Novel approaches have emerged to alleviate xerostomia and enhance salivary secretion in diabetic patients. Wu et al. reported that sialography‐guided injection of pancreatic lipase into the salivary glands can effectively mitigate diabetes‐related chronic obstructive parotitis.^169^ Additionally, Muhamed et al. confirmed the efficacy of a topical saliva stimulant spray containing 1% malic acid for treating xerostomia in patients with T2DM.^170^ Furthermore, animal studies have demonstrated that both atropine and metformin can ameliorate salivary gland damage in diabetic rats and alleviate symptoms of xerostomia, with effects being particularly pronounced when used in combination.^171^ These findings may offer new insights and strategies for the clinical management of diabetic xerostomia.

### 2.7 Diabetes and dental caries

Diabetic patients exhibit a heightened risk of developing dental caries,^172^ which is influenced by their glycemic control and the duration of diabetes.^173^ Two meta‐analyses have explored the relationship between diabetes and dental caries. One analysis revealed that the overall prevalence of dental caries in children and adolescents with type 1 diabetes was 67%, with the highest prevalence observed in South America and the lowest among patients maintaining good metabolic control.^174^ The other study indicated that type 2 diabetes may contribute to an increase in the incidence of caries among adults.^50^

The mechanisms underlying the association between diabetes and dental caries remain inadequately explored; however, they may be related to the effects of diabetes on saliva flow rate and buffering capacity, as well as alterations in the oral microbiota of affected individuals. Research has demonstrated that individuals with diabetes exhibit elevated levels of Lactobacillus alongside diminished saliva flow and buffering capacity. These factors facilitate the proliferation of *Streptococcus mutans*, thereby increasing the risk of dental caries.^175^, ^176^

### 2.8 Diabetes and root fracture

Root fracture refers to a break or crack in the root of a tooth. The incidence of vertical root fracture (VRF) in diabetic patients is 2.67 times higher than that observed in nondiabetic individuals.^177^ Furthermore, the resistance to root fracture in the premolars of diabetic patients is significantly lower compared to their nondiabetic counterparts.^178^

The impact of diabetes on the physiological structure of teeth may be related to the increased incidence of root fracture in diabetic patients. Research has found that diabetes can disrupt enamel development, leading to alterations in its microstructure and mechanical properties.^179^ Additionally, diabetes affects the nanostructure of dentin, with concentrations of elements such as magnesium, zinc, strontium, lithium, manganese, and selenium being significantly lower than those in nondiabetic patients, while copper concentrations are higher in diabetic patients.^180^

## 3 RHEUMATOID ARTHRITIS

RA is a chronic systemic autoimmune disorder that primarily targets the joints, resulting in pain, stiffness, and a decline in joint function.^181^ Patients with RA exhibit a heightened incidence of dental caries, pulpitis, gingivitis, periodontitis, and oral ulcers.^182^ The oral health‐related quality of life (OHRQoL) in patients with RA is compromised, influenced by factors such as the type of RA and the severity of periodontitis.^183^, ^184^ Consequently, understanding the relationship between RA and oral health, along with the underlying mechanisms, is crucial for the prevention and management of oral diseases in RA patients and may contribute to enhanced overall health outcomes.

To provide readers with a concise overview of the connections between RA and oral diseases, we summarize recent findings from relevant clinical studies in the table, highlighting key factors involved (Table 3).

**TABLE 3:** Clinical trials related to rheumatoid arthritis (RA) and oral health.

Diseases	Type of study	Aim	Sample	Sample	Molecule	Conclusions
Periodontitis	Cross‐sectional study	Evaluate the relationship between periodontal parameters and the presence and levels of ACPAs.	164 RA patients	Blood	ACPA	In RA, the severity of periodontal conditions is linearly correlated with the presence and levels of ACPAs.^185^
	Retrospective cross‐sectional study	Assess the production of NETs in patients with periodontitis and RA and their relationship with clinical parameters.	Early RA (*n* = 24), established RA (*n* = 64) and individuals without RA (*n* = 76)	Saliva and plasma	NETs	NETs may link PD and RA, with periodontal treatment significantly altering circulating NET levels.^186^
	Cross‐sectional study	Investigate whether serum levels of TREM‐1 and PGLYRP1 are associated with periodontitis in patients with RA.	154 participants with RA (*n* = 55), Behcet's disease (*n* = 41) and healthy controls (*n* = 58)	Serum and saliva	TREM‐1 and PGLYRP1	Elevated serum TREM‐1 is associated with PD and disease activity in RA.^187^
	Cross‐sectional study	Examine the effects of periodontitis and RA on the serum and saliva concentrations of MCP‐1, MIF, and fractalkine.	Periodontitis (*n* = 21), or with RA (*n* = 23), or with both diseases (*n* = 23), systemically and periodontally healthy individuals (*n* = 22)	Saliva and serum	MCP‐1, MIF, fractalkine	In RA, saliva shows elevated levels of MCP‐1, MIF, and fractalkine.^188^
	Case–control study	Investigate the genetic variations of PTPN22, PADI4, and CTLA4 and their impacts on RA and periodontitis.	111 RA patients and 256 systemically healthy controls	Blood, plaque	PTPN22, PADI4, CTLA4	Genetic variations may suggest a potential link between periodontitis and RA.^189^
	Cross‐sectional study	Investigate salivary and serum IgA ACPA in a population‐based cohort of elderly RA patients.	Patients with RA ≥ 61 years of age (*n* = 132)	Serum and saliva	Serum IgG ACPA and serum and saliva IgA ACPA	Serum and salivary IgA ACPA are not commonly found in RA patients with PD.^190^
	Retrospective cohort study	Explore the relationship between periodontal and serological indicators and disease activity in RA.	127 patients with RA	Serum	Anti‐agalactosyl immunoglobulin G	PF is positively correlated with anti‐agalactosyl IgG titers, and both show a positive association with disease activity in RA.^191^
	Cross‐sectional study	Investigate the relationship between antibody titers against fibrinogen‐derived CCP and anti‐CEP‐1 in RA patients.	107 patients with RA	Plasma and plaque	Anti‐CCP and anti‐CEP‐1 antibodies	In RA, PD may be a secondary risk factor for anti‐CCP positivity.^192^
	Case–control study	Investigate whether the association between periodontitis and RA is influenced by SNPs in the genes encoding PAD2 and PAD4.	137 RA patients and 161 controls with self‐reported periodontitis	Blood	rs2057094、rs2076616、rs2235912	Carriers of the alleles for SNPs rs2057094, rs2076616, and rs2235912 in the PADI2 gene may have an increased risk of developing RA in patients with periodontitis.^193^
	Case–control study	Quantify the relationship between periodontal clinical indicators, ABL, concentrations of antibodies against *Porphyromonas gingivalis* (*P. gingivalis*), *P. intermedia*, and *Fusobacterium nucleatum* (*F. nucleatum*), and the concentration of anti‐MAA antibodies.	(*n* = 284 RA cases, *n* = 330 OA controls)	Plasma	anti‐MAA antibodies	MAA may play a role in the interaction between periodontal tissues and RA.^194^
	Case‐controlled clinical trial	Investigate the levels of PRL in GCF, synovial fluid, and serum of patients with moderate active RA, both with and without periodontitis.	80	GCF, synovial fluid, and plasma	PRL	Local PRL levels in GCF and synovial fluid seem to be associated with the disease processes of periodontitis and RA, compared to serum levels.^195^
Sjögren's syndrome	Cross‐sectional research	Investigate the relationship between NLRP3 gene polymorphisms and patients with RA and primary SS.	A total of 239 patients with RA, 285 patients with primary SS, and 170 controls	Blood	NLRP3 gene polymorphism	The NLRP3 genotype may affect clinical outcomes and progression in RA and primary SS. The activity of the NLRP3 inflammasome may explain the severity of these diseases.^196^

Abbreviations: ABL, alveolar bone loss; ACPA, anti‐citrullinated protein antibody; anti‐CCP, anti‐cyclic citrullinated peptides; anti‐CEP‐1, anti‐citrullinated α‐enolase peptides; ARE, arylesterase; CMV, cytomegalovirus; EBV, Epstein–Barr virus; GCF, gingival crevicular fluid; MAA, malondialdehyde‐acetaldehyde; MCP‐1, monocyte chemoattractant protein‐1; MIF, migration inhibitory factor; NETs, neutrophil extracellular traps; OSI, oxidative stress index; PD, periodontitis; PGLYRP1, peptidoglycan recognition protein 1; PRL, prolactin; SNP, single nucleotide polymorphism; SS, Sjögren's syndrome; TAS, total antioxidant status; TMD, temporomandibular disorders; TOS, total oxidative status; TREM‐1, triggering receptor expressed on myeloid cells 1.

### 3.1 RA and periodontitis

**FIGURE 3:**
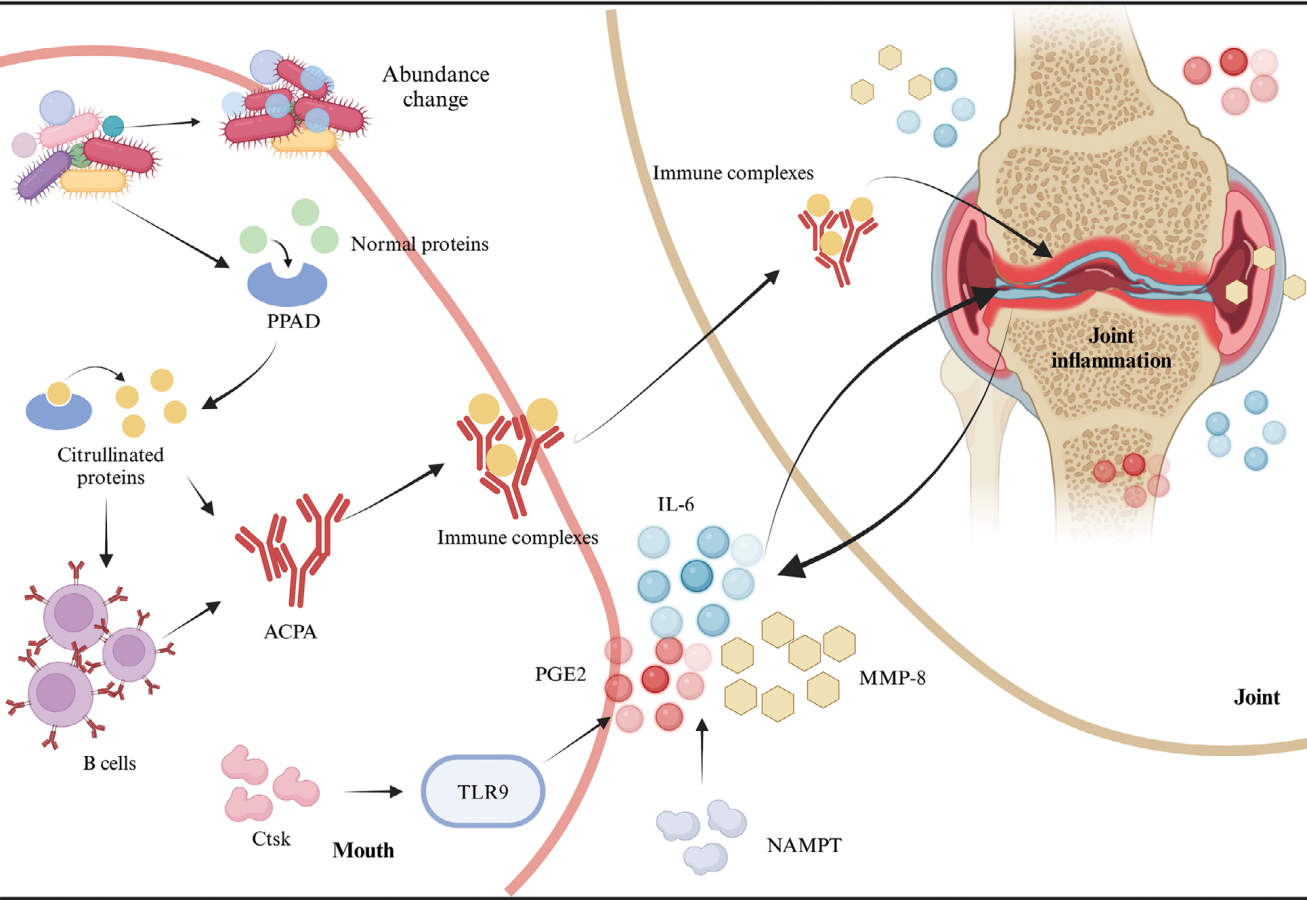
Mechanisms linking oral health and rheumatoid arthritis. Microorganisms such as *Porphyromonas gingivalis* (*P. gingivalis*) produce PPAD, which citrullinates proteins and stimulates B cells to generate ACPAs. These ACPAs form immune complexes that accumulate in the joints, contributing to the pathogenesis of rheumatoid arthritis. Additionally, NAMPT and Ctsk trigger inflammatory responses through distinct pathways, elevating levels of inflammatory cytokines and MMP‐8, ultimately leading to joint tissue damage. Furthermore, the composition of oral bacteria is altered in patients with rheumatoid arthritis. ACPA, anti‐citrullinated protein antibodies; Ctsk, cathepsin K; IL‐6, interleukin‐6; MMP‐8, matrix metalloproteinase 8; NAMPT, nicotinamide phosphoribosyl transferase; PGE2, prostaglandin E2; PPAD, *P. gingivalis* peptidyl arginine deiminase. Created with BioRender.com.

Over the past decade, numerous studies worldwide have confirmed the correlation between RA and periodontitis.^197^, ^198^, ^199^, ^200^ On one hand, certain RA medications appear to elevate the risk of periodontal inflammation and exacerbate periodontitis.^201^, ^202^ Conversely, periodontal treatment has been shown to reduce RA activity.^181^, ^203^, ^204^ The mechanisms by which RA influences periodontitis primarily involve microbial factors, inflammatory mediators, and genetic associations (Figure 3).

Patients with periodontitis and RA often exhibit an abnormal abundance of specific microorganisms,^205^, ^206^ along with limited variation in microbial taxa and gene functions between deep and shallow subgingival sites.^207^ These findings suggest that RA may influence the development and progression of periodontitis through alterations in the microbiome. Furthermore, specific oral pathogens such as *Porphyromonas gingivalis* (*P. gingivalis*) produce peptidyl arginine deiminase (PPAD),^208^ which can citrullinate proteins, resulting in the formation of citrullinated autogenic antigens (CPAs) and subsequently leading to the production of anti‐citrullinated protein antibodies (ACPAs).^209^

Furthermore, RA and periodontitis share common molecular mediators, such as MMP‐8, IL‐6, and prostaglandin E2.^210^, ^211^ These same mediators may play a role in their interaction. RA can also influence periodontitis by regulating inflammatory factors through various upstream mediators. Studies have shown that nicotinamide phosphoribosyl transferase (NAMPT) expression is upregulated in the periodontal ligament tissues of RA mice, resulting in elevated levels of proinflammatory cytokines (IL‐6), chemokines (IL‐8, CCL5), and inflammatory mediators such as COX‐2, MMP‐1, and MMP‐3 in periodontal ligament cells.^212^ Another study suggests that cathepsin K (Ctsk) influences the infiltration of dendritic cells and T cells, as well as the production of inflammatory factors through the TLR9 signaling pathway, serving as a key pathological regulator in both RA and periodontitis. This mechanism contributes to enhanced erosion of periodontal bone and knee cartilage.^213^

In addition to the aforementioned microbial and inflammatory factors, other studies have explored genetic associations. It was found that IL‐1α‐889 C/IL‐1β‐511a is positively correlated with RA.^214^ Furthermore, another study indicated that a high incidence and severity of periodontitis in first‐degree relatives (FDRs) of RA patients are associated with seropositivity for ACPAs, further supporting the hypothesis that periodontitis may serve as a risk factor for the development of RA.^215^

### 3.2 RA and other oral diseases

#### 3.2.1 Temporomandibular disorders

Many patients with RA exhibit clinical manifestations of temporomandibular joint (TMJ) inflammation, with the most common symptoms including pain in the TMJ area during movement or stress, joint clicking, and impaired mandibular function.^216^ A large‐scale clinical study in Taiwan found that the risk of temporomandibular disorders (TMD) in patients with RA is 2.538 times higher than that in non‐RA patients.^217^ Bone resorption occurs within the TMJ of RA patients, potentially mediated by TNF through synovial fluid.^218^ In terms of treatment, animal studies have demonstrated that bone marrow mesenchymal stem cells (BMSCs) significantly enhance the healing of TMJs in rats with induced RA.^219^

#### 3.2.2 Sjögren syndrome and xerostomia

It has been reported that 51% of patients with RA experience dry mouth, and a statistically significant relationship exists between dry mouth and poor OHRQoL. The rates of whole‐mouth saliva at rest and stimulated parotid saliva flow in RA patients are significantly lower than those observed in non‐RA individuals.^220^ The OHRQoL of RA patients is adversely affected by dry mouth syndrome, which subsequently impacts their overall quality of life.^184^ Regarding the underlying mechanism, a case–control study was conducted to investigate the role of viruses in the association between RA and Sjögren's syndrome (SS), revealing that Epstein–Barr virus (EBV) is more prevalent among RA patients and correlates with Schirmer test results.^221^

#### 3.2.3 Dental caries and apical periodontitis

It has been observed that patients with RA exhibit a higher frequency and severity of dental plaque, with significantly elevated counts of *S. mutans* in the RA group.^222^ There may also be an association between increased levels of systemic inflammatory cytokines induced by RA and AP. A cross‐sectional study demonstrated that RA is significantly associated with a higher prevalence of AP, indicating that RA patients are more likely to develop this condition. However, RA does not appear to influence the outcomes of RCT.^223^

## 4 CARDIOVASCULAR DISEASES

CVDs, encompassing conditions such as ischemic heart disease, stroke, heart failure, peripheral artery disease, and various other cardiac and vascular disorders, represent a leading cause of mortality worldwide and significantly impair quality of life.^224^, ^225^ In 2019, CVDs accounted for 33% of all global deaths, with ischemic heart disease (9.1 million deaths) and stroke (6.6 million deaths) collectively representing 85% of fatalities related to CVDs.^226^ There is a strong association between CVDs and oral health, particularly through the link between atherosclerosis and periodontitis. Additionally, an interplay exists between hypertension and periodontitis. Furthermore, patients with CVDs frequently exhibit higher rates of dental caries and tooth loss.

Consequently, it is imperative to underscore the critical role of clinical practitioners, dental professionals, and other healthcare providers in comprehending the relationship between periodontal disease and CVDs, identifying their risk factors, and recognizing the urgent need for timely referrals to specialized dental or periodontal care. Mitigating the prevalence and incidence of periodontal disease may contribute to a reduction in the associated risks of systemic diseases.

### 4.1 Atherosclerotic vascular disease and periodontal diseases

Atherosclerotic vascular disease (ASVD) is a chronic condition characterized by the accumulation of lipids, cholesterol, and other substances within the arterial walls, leading to the formation of atherosclerotic plaques.^227^ There is a significant increase in risk associated with chronic periodontitis and ASVD, independent of other established cardiovascular risk factors.^228^ Meta‐analyses encompassing prospective cohort studies, case–control studies, and cross‐sectional studies consistently demonstrate a markedly elevated risk of coronary heart disease (CHD) in patients with periodontal disease.^7^ Furthermore, periodontal disease increases the incidence of myocardial infarction by two to four times,^229^ while severe periodontal disease can elevate the risk of stroke by 3.5%.^230^

**FIGURE 4:**
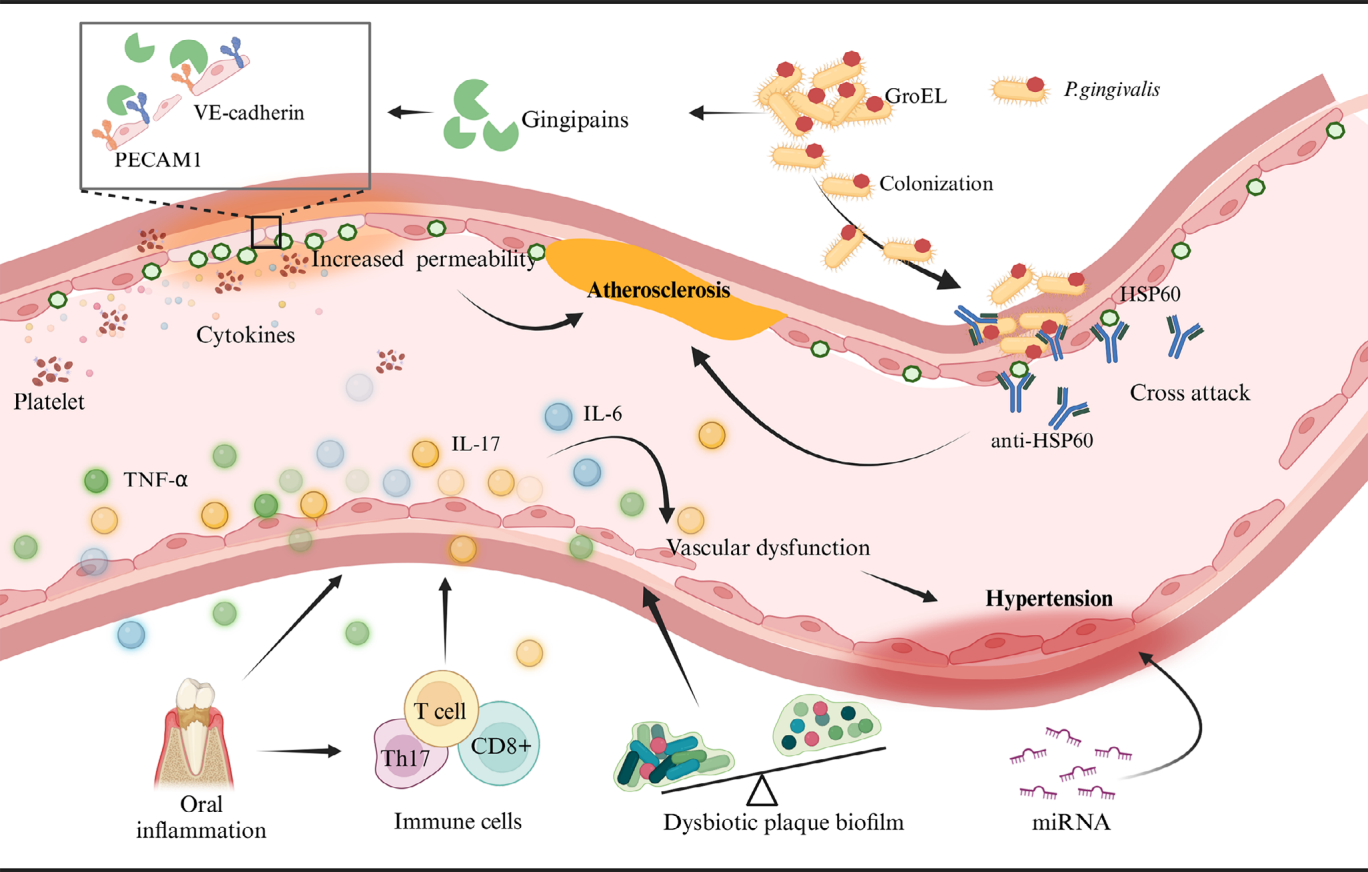
Mechanisms linking oral health and cardiovascular diseases. **Atherosclerosis**: *Porphyromonas gingivalis* (*P. gingivalis*) contains GroEL protein, which shares structural similarities with the HSP60 epitopes expressed by vascular endothelial cells. Consequently, antibodies against HSP60 may inadvertently target and damage these endothelial cells, thereby promoting the development of atherosclerosis. Additionally, *P. gingivalis* produces gingipains that degrade PECAM‐1 and VE‐cadherin, disrupting the integrity of the endothelial barrier, increasing permeability, promoting platelet aggregation, and triggering the release of proinflammatory cytokines—factors that may accelerate the progression of atherosclerosis. Created with BioRender.com. **Hypertension**: The inflammatory environment within the oral cavity releases cytokines such as TNF‐α, IL‐6, and IL‐17, which can impair endothelial function and contribute to hypertension. Furthermore, this inflammatory milieu activates various immune cells, exacerbating cytokine release and resulting in vascular dysfunction. Additionally, dysbiosis of the oral microbiota may also be associated with hypertension. HSP60, heat shock protein 60; IL‐6, interleukin‐6; IL‐17, interleukin‐17; PECAM1, platelet endothelial cell adhesion molecule; TNF‐α, tumor necrosis factor‐alpha; VE‐cadherin, vascular endothelial cadherin; WBC, white blood cell. Created with BioRender.com.

The underlying mechanisms linking ASVD and periodontitis primarily involve infection, molecular mimicry, and systemic inflammation (Figure 4).^231^ Furthermore, numerous lines of evidence suggest a potential association between *P. gingivalis* and atherosclerotic diseases related to periodontal disease. First, *P. gingivalis* can invade cardiovascular cells,^232^ with its distant invasion correlating with an increased risk of acute myocardial infarction,^233^ indicating that it may exacerbate the severity of coronary artery disease through the induction of systemic inflammation. Second, *P. gingivalis* degrades platelet endothelial cell adhesion molecules (PECAM‐1) and vascular endothelial cadherin (VE‐cadherin), compromising the integrity of the endothelial barrier and enhancing vascular permeability.^234^ This disruption may promote platelet aggregation and the release of proinflammatory cytokines, potentially accelerating the progression of atherosclerosis.^235^ Additionally, the presence of *P. gingivalis* is often associated with significantly elevated levels of antibodies against human heat shock protein 60 (HSP60).^236^ Under conditions characterized by endothelial dysfunction, HSP60 expression is observed; its epitopes share similarities with GroEL protein from *P. gingivalis*,^237^ which may lead to cross‐reactivity that further exacerbates endothelial damage and accelerates atherosclerosis.

### 4.2 Hypertension and periodontal diseases

Hypertension is a prevalent risk factor for CVDs, affecting approximately 45% of the global population, with prevalence rates increasing with age.^238^ Notably, about 50% of hypertensive patients fail to achieve effective blood pressure control.^239^, ^240^ This high prevalence can largely be attributed to a lack of public awareness regarding the classic triggers of hyperadherence to treatment recommendations among patients.^241^ However, it is crucial to consider that nonclassical cardiovascular risk factors, such as periodontitis, may also contribute to the onset and poor management of hypertension.^242^

Clinical data and systematic reviews indicate that patients with periodontal disease exhibit significantly higher systolic and diastolic blood pressure compared to those without the condition,^243^ with a notable association between moderate to severe periodontal disease and an increased risk of hypertension.^244^ Furthermore, there is a significant correlation among periodontal disease, the number of missing teeth, and the experience of basic periodontal treatment concerning future incidence of hypertension.^245^ Despite considerable heterogeneity across studies, current evidence suggests that periodontal treatment is associated with a marked reduction in C‐reactive protein (CRP) levels and an improvement in endothelial function, along with varying degrees of blood pressure reduction.^11^, ^246^, ^247^

The pathogenesis of hypertension in patients with periodontitis is complex and not yet fully elucidated, involving multiple interconnected mechanisms. For instance, inflammatory responses characterized by elevated levels of inflammatory molecules during periodontitis impair endothelial function and contribute to increased blood pressure (Figure 4).^241^, ^248^, ^249^ Immune cell responses are also significant; activated T cells migrate to vascular tissues, releasing proinflammatory cytokines that lead to vascular dysfunction.^249^, ^250^, ^251^ Additionally, microbial dysbiosis in the oral cavity, marked by pathogenic bacteria associated with periodontitis, correlates with elevated blood pressure.^252^, ^253^, ^254^ Finally, noncoding RNAs, particularly microRNAs, may serve as epigenetic regulators linking periodontitis and hypertension.^255^

### 4.3 Other oral diseases

In addition to periodontitis, patients with CVDs demonstrate a higher incidence of dental caries and tooth loss.^228^, ^256^, ^257^ The loss of five or more teeth is significantly associated with an increased risk of CHD events and acute myocardial infarction. When the number of missing teeth reaches nine or more, there is a notable association with the risk of CVDs, diabetes, and all‐cause mortality.^258^ A meta‐analysis revealed a linear relationship between tooth loss and CHD mortality.^259^

## 5 CHRONIC RESPIRATORY DISEASES

The primary function of the respiratory system is to facilitate the exchange of oxygen and carbon dioxide, thereby supporting the metabolic activities of the body's cells. Common respiratory diseases include chronic obstructive pulmonary disease (COPD), asthma, and pneumonia. According to the 2019 Global Burden of Disease study, chronic respiratory diseases ranked as the third leading cause of death, accounting for 4 million fatalities worldwide and affecting approximately 454.6 million cases (ranging from 417.4 to 499.1 million).^260^

The oral cavity is anatomically connected to the upper respiratory tract, and as air and food traverse the mouth into the pharynx, pathogens or inflammatory substances may enter the throat or lungs, potentially triggering or exacerbating respiratory diseases such as pneumonia, COPD, asthma, and lung cancer.^261^ Studies have demonstrated a significant degree of similarity between the oral and lung microbiomes in healthy individuals.^262^

Research shows that oral diseases are closely associated with various lung diseases, such as asthma, COPD, pulmonary fibrosis, and pneumonia.^263^, ^264^ Patients with chronic respiratory diseases show a declining trend in OHRQoL.^265^

### 5.1 COPD

Patients with COPD often exhibit poorer oral health, as evidenced by worse periodontal status, higher plaque indices, and elevated DMFT (decayed, missing, filled teeth) scores.^266^ Poor oral conditions or diseases, such as periodontitis,^267^, ^268^ dental caries,^264^ and tooth loss^269^ can exacerbate airflow limitation in COPD patients, worsening daily respiratory symptoms like coughing and wheezing and potentially contributing to acute exacerbations that lead to increased hospitalization rates and higher mortality. Direct evidence demonstrating that oral care interventions can improve outcomes in COPD is currently limited; however, two small‐scale trials have suggested that periodontal treatment may reduce the frequency of COPD exacerbations.^12^, ^270^

**FIGURE 5:**
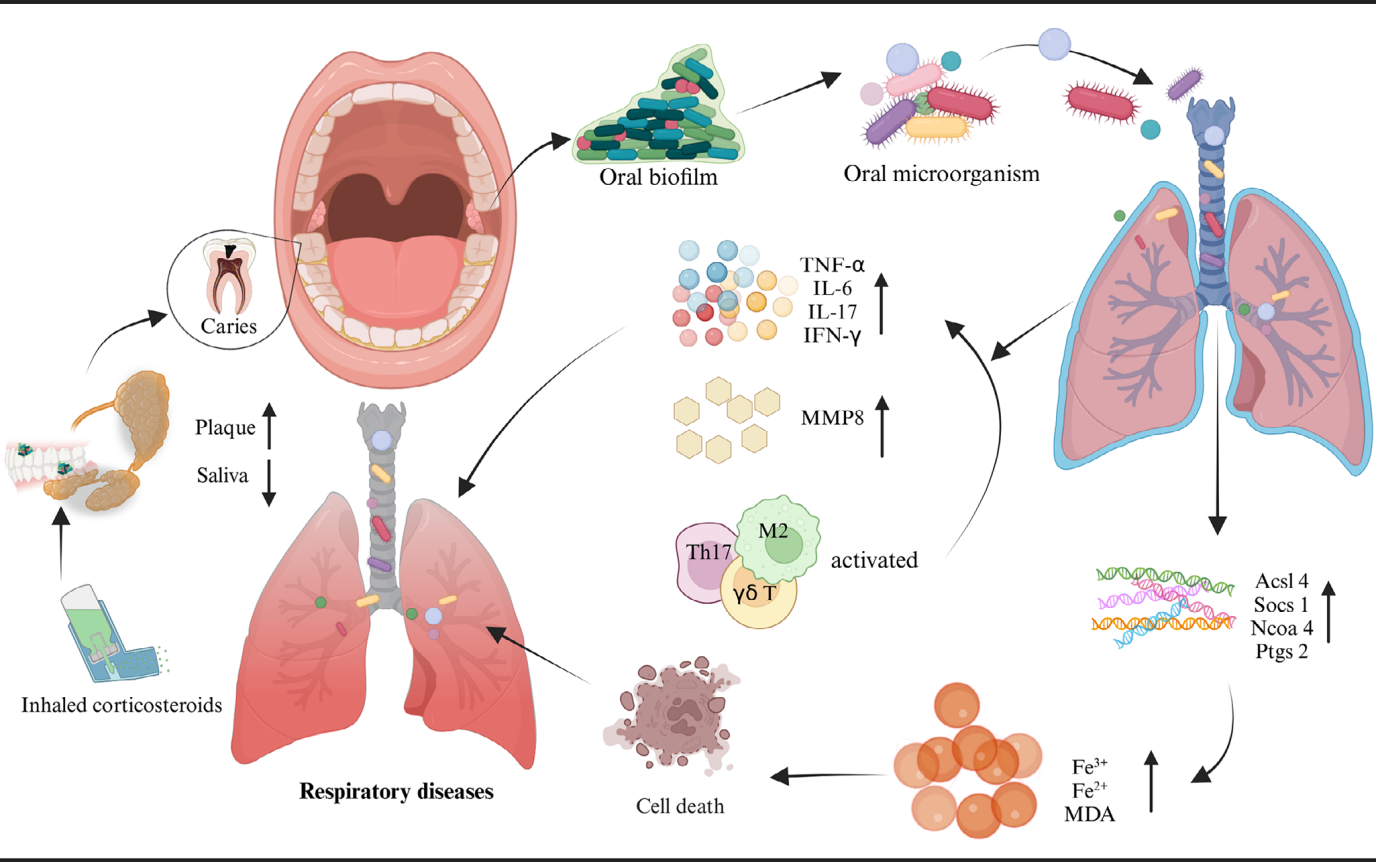
Mechanisms linking oral health and chronic respiratory diseases. Under specific conditions, oral bacteria can translocate to the respiratory tract and colonize the lungs. This may trigger immune and inflammatory responses, leading to the production of cytokines and MMPs that can damage lung tissue, potentially impacting oral tissues as well. Additionally, the expression of ferroptosis‐promoting genes such as Acsl4, Socs1, Ncoa4, and Ptgs2 are upregulated, inducing ferroptosis and contributing to chronic respiratory diseases. Furthermore, treatments for chronic respiratory diseases—such as inhaled corticosteroids—may increase plaque formation and reduce salivary flow rate, thereby elevating the risk of dental caries. Acsl4/Socs1/Ncoa4/Ptgs2, ferroptosis‐related genes; IFN‐γ, interferon‐gamma; IL‐6, interleukin‐6; IL‐17, interleukin‐17; MDA, malondialdehyde; MMP‐8, matrix metalloproteinase 8; TNF‐α, tumor necrosis factor‐alpha. Created with BioRender.com.

Research on the mechanisms linking COPD and various oral diseases is limited, with many studies conducted using animal models. These mechanisms may involve immune responses, microbial factors, and ferroptosis (Figure 5). Mutual exacerbation of periodontitis and COPD is associated with the activation of γδ T cells and M2 macrophages, ultimately leading to increased expression of IL‐17 and interferon gamma (IFN‐γ), as well as M2 macrophage polarization.^271^ Furthermore, *P. gingivalis* or Fusobacterium nucleatum (*F. nucleatum*) promoted the development of COPD and impaired lung function in mice.^272^ Studies have also shown that periodontitis accelerates the progression of COPD by upregulating ferroptosis in lung tissue.^273^

### 5.2 Bronchial asthma (asthma)

Patients with asthma exhibit altered salivary flow rates and composition, as well as poorer oral health, characterized by a higher prevalence of dental caries.^274^, ^275^ Medications, such as inhaled corticosteroid (ICS) therapy used to treat bronchial asthma are also associated with an increased incidence of dental caries.^276^ The heightened risk of dental caries linked to ICS may be attributed to changes in salivary composition and flow rate.^277^ Furthermore, clinical data and systematic reviews indicate that asthma patients may have an elevated risk of periodontitis^278^, ^279^ and tooth loss.^280^

Currently, there is no conclusive evidence establishing a direct biological link between periodontitis and asthma. The prevailing theoretical framework posits that these two inflammatory diseases may influence one another through microbial interactions, inflammatory factors, and immune responses (Figure 5). Research has shown that the periodontal pathogen *Prevotella* can induce the production of matrix metalloproteinases (MMPs), which adversely affect both periodontitis and asthma.^281^ Additionally, MMPs can be produced by various inflammatory cells in the respiratory tract, potentially exacerbating periodontal inflammation.^282^ On the immunological front, periodontal pathogens activate host immune cells to produce cytokines such as prostaglandin E2 (PGE2), interferon‐γ, TNF‐α, and several interleukins (IL‐1, IL‐6, IL‐10, IL‐11). These cytokines stimulate macrophages and osteoclasts to release hydrolases and collagenases—enzymes that degrade collagen and elastic fibers in lung tissue—thereby contributing to bronchial remodeling processes in asthma.^283^ Moreover, Th17 cells are implicated in various immune‐mediated inflammatory conditions such as psoriasis and RA; they serve as potent mediators of tissue inflammation. Interleukin 17A (IL‐17A) can activate a range of inflammatory cascades that mediate the development and progression of periodontitis along with related systemic chronic inflammatory diseases like asthma.^284^

### 5.3 Pneumonia

Epidemiological studies have demonstrated a correlation between oral health issues and the risk of developing pneumonia. Data from the Korean national population indicate that oral health problems, such as dental caries and tooth loss, significantly increase the risk of pneumonia; frequent tooth brushing and regular professional dental cleanings are associated with a reduced incidence of this condition.^285^ In specific populations, including intensive care unit (ICU) patients,^286^ adults with severe and complex neurological disorders,^287^ postoperative cancer patients,^288^ and acute stroke patients,^289^ professional oral care has been linked to a decrease in pneumonia incidence. When examining specific types of pneumonia, evidence supporting the link between oral health is more consistent for hospital‐acquired pneumonia (HAP),^290^, ^291^ whereas the association with community‐acquired pneumonia (CAP) remains controversial.^292^, ^293^, ^294^, ^295^

Regarding the potential mechanisms linking oral health to the development of various types of pneumonia, Kim et al. proposed that the oral environment can serve as a reservoir for pneumonia pathogens, which may be aspirated into the lower respiratory tract under certain conditions, thereby creating a favorable environment for pneumonia development.^293^ Recent studies have reaffirmed that oral bacteria can act as a source of infection in aspiration pneumonia, with the quantity of oral bacteria identified as a significant risk factor for this condition.^296^, ^297^ Furthermore, inflammatory cytokines induced by oral inflammatory diseases such as periodontitis play a crucial role in pneumonia pathogenesis.^298^ Recent animal experiments have demonstrated that *P. gingivalis* induces pneumonia in mice with periodontitis, significantly increasing levels of cytokines and neutrophils in peripheral blood and lung tissue.^299^ A recent study innovatively transplanted human oral commensal microbiota into germ‐free mice to create human oral microbiota‐associated (HOMA) mice; this study analyzed the impact of oral microbiota on lung immunity and systemic conditions during acute lung injury and acute respiratory distress syndrome (ALI/ARDS).^300^ The results indicated that HOMA mice exhibited systemic dysbiosis; compared to conventional (CNV) mice with sufficient microbial diversity, HOMA mice developed more severe ALI.

Mouthwash is a critical component of oral hygiene, and chlorhexidine mouthwash has been shown to reduce the incidence of respiratory infections; however, long‐term use may result in side effects including altered taste, dry mouth, and tooth discoloration. Over the past decade, research has increasingly focused on exploring new mouthwashes derived from natural products. A study comparing a 6.66% clove extract mouthwash with a 0.2% chlorhexidine mouthwash demonstrated that the control group had a 2.06‐fold higher risk of developing ventilator‐associated pneumonia (VAP) than the intervention group.^13^ Additionally, a triple‐blind randomized controlled trial investigating the effects of propolis mouthwash revealed that the intervention group experienced significantly lower incidences of VAP on days 3, 5, and 7 compared to the control group. Propolis mouthwash may represent a viable alternative to chlorhexidine for patients in ICU.^301^

## 6 CONCLUSION

Chronic diseases and oral health are intricately linked, as conditions such as periodontitis, dental caries, and AP can potentially increase the risk of chronic illnesses through pathways like systemic inflammation and pathogen transmission. Addressing oral hygiene is not only crucial for dental health but also plays a significant role in reducing the burden of chronic diseases. Improving oral health management, raising public awareness, and providing accessible and cost‐effective dental care can have a profound impact on public health outcomes. Medical institutions should consider integrating oral health into comprehensive patient care for individuals with CVDs, particularly during rehabilitation, by incorporating oral health education and preventive care. This holistic approach could mitigate long‐term risks while enhancing the overall quality of life for these patients.

The intricate and intimate connection between chronic diseases and oral health offers an extensive array of exploration prospects for future research and clinical practice. Future research, for the first, will focus on uncovering the underlying mechanisms of these interactions, with the goal of identifying new therapeutic targets. For example, mechanistic studies on diabetes and periodontitis suggest future drug targets may involve proresolving pathways, host response modulation, Th17/Treg imbalance, antioxidant therapy, immune training, and genetic modifications.^302^, ^303^ The integration of emerging technologies, such as multiomics approaches and artificial intelligence (AI), will be crucial for discovering new mechanism.^304^, ^305^ In the future, noninvasive diagnostic tools, such as saliva‐based rapid detection and continuous monitoring devices, will enable early detection and ongoing management of oral health issues linked to chronic diseases.^306^, ^307^

## AUTHOR CONTRIBUTIONS

**Di Fu**: conception of the work; interpretation of data for the work; drafting the work. **Xingyue Shu**: conception and design of the work; Substantial contributions to acquisition and analysis of data for the work. **Ge Zhou**: revised the manuscript. **Mengzhen Ji**: revised the manuscript. **Ga Liao**: contributions to acquisition and analysis of data for the work; revised the manuscript critically for important intellectual content. **Ling Zou**: conception and design of the work; revising it critically for important intellectual content; acquisition of fundings. All the authors have read and approved the final manuscript.

## CONFLICT OF INTEREST STATEMENT

The authors declare no conflicts of interest.

## ETHICS STATEMENT

Not applicable.

## Data Availability

Data availability is not applicable to this review as no new data were created or analyzed in this review.
